# Predictive Value of Post-Percutaneous Coronary Intervention Quantitative Flow Ratio for Vessel-Oriented Composite Endpoint

**DOI:** 10.1155/2023/2438347

**Published:** 2023-09-09

**Authors:** Weibin Liu, Huaxiu Cai, Yin Zheng, Yongkang Wen, Sicheng Chen, Xiuying Xie, Huan Zeng, Hengqing Zhu, Zhonghan Ni, Fang Pei, Jun Cao, Gang Cao

**Affiliations:** ^1^Department of Cardiology, Ganzhou People's Hospital, Ganzhou 341000, China; ^2^Gannan Medical University, Ganzhou 341000, China; ^3^Department of General Practice, Ganzhou Hospital of Guangdong Provincial People's Hospital, Ganzhou Municipal Hospital, Ganzhou 341000, China; ^4^Department of Radiology and Imaging, Ganzhou Hospital of Guangdong Provincial People's Hospital, Ganzhou Municipal Hospital, Ganzhou 341000, China; ^5^Department of Cardiology, Ganzhou Hospital of Guangdong Provincial People's Hospital, Ganzhou Municipal Hospital, Ganzhou 341000, China

## Abstract

At present, there is a lack of indicators, which can accurately predict the post-percutaneous coronary intervention (post-PCI) vessel-oriented composite endpoint (VOCE). Recent studies showed that the post-PCI quantitative flow ratio (QFR) can predict post-PCI VOCE. PubMed, Embase, and Cochrane were searched from inception to March 27, 2022, and the cohort studies about that the post-PCI QFR predicts post-PCI VOCE were screened. Meta-analysis was performed, including 6 studies involving 4518 target vessels. The results of the studies included in this meta-analysis all showed that low post-PCI QFR was an independent risk factor for post-PCI VOCE after adjusting for other factors, HR (95% CI) ranging from 2.718 (1.347–5.486) to 6.53 (2.70–15.8). Our meta-analysis showed that the risk of post-PCI VOCE was significantly higher in the lower post-PCI QFR group than in the higher post-PCI QFR group (HR: 4.14, 95% CI: 3.00–5.70, *P* < 0.001, *I*^2^ = 27.9%). Post-PCI QFR has a good predictive value for post-PCI VOCE. Trial Registration. This trial is registered with CRD42022322001.

## 1. Introduction

Coronary atherosclerotic heart disease (CHD) is one of the most common cardiovascular diseases in clinical practice. Medication and percutaneous coronary intervention (PCI) can relieve symptoms and improve outcome in patients with CHD. However, some patients may still experience vessel-oriented composite endpoint (VOCE), defined as the composite of vessel-related cardiac death, vessel-related myocardial infarction, and target vessel revascularization. The incidence of post-PCI VOCE was about 7% [1, 2]. Previous studies have shown that intravascular ultrasound (IVUS), optical coherence tomography (OCT), and fractional flow reserve (FFR) can predict the long-term prognosis of the post-PCI patients [3–10]. However, these methods exert some limitations, such as invasive, high cost, and complex operation. Therefore, finding a simple method that can accurately predict the long-term prognosis of the post-PCI patients is crucial.

In 2015, Tu [11] proposed a quantitative flow ratio (QFR) based on FFR, a noninvasive and guidewire-free FFR rapid analysis system. The QFR combines a three-dimensional reconstruction technology of coronary angiography, a hemodynamic system, and artificial intelligence with respect to blood flow quantification to accurately evaluate the physiological function of the coronary artery.

Recently, some studies have shown that post-PCI QFR can predict post-PCI VOCE [1, 12–19]. Among them, the results of 6 cohort studies showed that the risk of post-PCI VOCE was higher in the group with lower post-PCI QFR than in the group with higher post-PCI QFR [1, 12–16]. However, the population and sample size of these 6 cohort studies were not exactly the same, and there were differences in hazard ratio (HR) values and 95% confidence interval (95% CI). Therefore, we conducted this meta-analysis to obtain the cumulative sample size and improve the statistical efficiency.

## 2. Materials and Methods

### 2.1. Search Strategy

This study was conducted according to the PRISMA statement. PubMed, Embase, and Cochrane were searched from inception to March 27, 2022, and the cohort studies about that the post-PCI QFR predicts post-PCI VOCE were screened. Subject words plus free words were used to search the PCI. Free words were used to search QFR, because QFR has no subject words. The expression of QFR was as follows: “QFR,” “quantitative flow ratio,” “Virtual FFR,” “virtual fractional flow ratio,” and “virtual pressure wire.” The search strategy of PubMed was as follows: (((“Percutaneous Coronary Intervention”[Mesh]) OR ((Coronary Intervention, Percutaneous^*∗*^) OR (Coronary Interventions, Percutaneous^*∗*^) OR (Intervention, Percutaneous Coronary^*∗*^) OR (Interventions, Percutaneous Coronary^*∗*^) OR (Percutaneous Coronary Interventions^*∗*^) OR (Percutaneous Coronary Revascularization^*∗*^) OR (Coronary Revascularization, Percutaneous^*∗*^) OR (Coronary Revascularizations, Percutaneous^*∗*^) OR (Percutaneous Coronary Revascularizations^*∗*^) OR (Revascularization, Percutaneous Coronary^*∗*^) OR (Revascularizations, Percutaneous Coronary^*∗*^)))) AND (((((QFR *∗* [Title/Abstract]) OR (quantitative flow ratio *∗* [Title/Abstract])) OR (Virtual FFR *∗* [Title/Abstract])) OR (virtual fractional flow ratio *∗* [Title/Abstract])) OR (virtual pressure wire *∗* [Title/Abstract])). The search strategy of Embase was as follows: (“percutaneous coronary intervention”/exp OR “coronary intervention, percutaneous^*∗*^”: ti, ab, kw OR “coronary interventions, percutaneous^*∗*^”: ti, ab, kw OR “intervention, percutaneous coronary^*∗*^”: ti, ab, kw OR “interventions, percutaneous coronary^*∗*^”: ti, ab, kw OR “percutaneous coronary interventions^*∗*^”: ti, ab, kw OR “percutaneous coronary revascularization^*∗*^”: ti, ab, kw OR “coronary revascularization, percutaneous^*∗*^”: ti, ab, kw OR “coronary revascularizations, percutaneous^*∗*^”: ti, ab, kw OR “percutaneous coronary revascularizations^*∗*^”: ti, ab, kw OR “revascularization, percutaneous coronary^*∗*^”: ti, ab, kw OR “revascularizations, percutaneous coronary^*∗*^”: ti, ab, kw) AND (“qfr^*∗*^”: ti, ab, kw OR “quantitative flow ratio^*∗*^”: ti, ab, kw OR “virtual ffr^*∗*^”: ti, ab, kw OR “virtual fractional flow ratio^*∗*^”: ti, ab, kw OR “virtual pressure wire^*∗*^”: ti, ab, kw). The search strategy of Cochrane was as follows: #1 = MeSH descriptor: [Percutaneous Coronary Intervention] explode all trees; #2 = (Coronary Intervention, Percutaneous^*∗*^) OR (Coronary Interventions, Percutaneous^*∗*^) OR (Intervention, Percutaneous Coronary^*∗*^) OR (Interventions, Percutaneous Coronary^*∗*^) OR (Percutaneous Coronary Interventions^*∗*^) OR (Percutaneous Coronary Revascularization^*∗*^) OR (Coronary Revascularization, Percutaneous^*∗*^) OR (Coronary Revascularizations, Percutaneous^*∗*^) OR (Percutaneous Coronary Revascularizations^*∗*^) OR (Revascularization, Percutaneous Coronary^*∗*^) OR (Revascularizations, Percutaneous Coronary^*∗*^); #3 = #1 OR #2; #4 = (QFR^*∗*^): ti, ab, kw OR (quantitative flow ratio^*∗*^): ti, ab, kw OR (Virtual FFR^*∗*^): ti, ab, kw OR (virtual fractional flow ratio^*∗*^): ti, ab, kw OR (virtual pressure wire^*∗*^): ti, ab, kw (Word variations have been searched); #5 = #3 AND #4. In addition, we manually searched two articles that met the criteria.

### 2.2. Inclusion Criteria and Exclusion Criteria

The inclusion criteria were as follows: (1) cohort study; (2) the study subjects were post-PCI CHD patients; (3) QFR of the target vessels was measured after successful PCI; (4) according to the post-PCI QFR, the target vessels of all patients together were divided into two cohorts: lower post-PCI QFR group and higher post-PCI QFR group. (5) The primary endpoint of the study was post-PCI VOCE. (6) Cox regression was used for multiple-variable analysis. (7) After adjusting for other influencing factors, post-PCI QFR was analyzed as an independent risk factor for post-PCI VOCE.

Case-control studies were cross-sectional and time-independent, in which odd ratio (OR) was generally used for statistical description. Outcome measures of cohort studies were time-dependent, in which hazard ratio (HR) was generally used for statistical description. It was not appropriate to put OR and HR together for statistical merging, and case-control studies had a lower level of evidence-based medical evidence than cohort studies. Therefore, we excluded 3 case-control studies [17–19].

### 2.3. Data Extraction

The data, such as author, year of publication, country, data source, follow-up time, number of cases, number of target vessels, age, the proportion of males, proportion of hypertension, proportion of diabetes, proportion of smoking, the proportion of hyperlipidemia, left ventricular ejection fraction, proportion of target vessels, PCI type, post-PCI QFR cutoff value, hazard ratio (HR), 95% confidence interval (95% CI), and number of post-PCI VOCE, were independently extracted by two researchers from the finally included studies. The risk of bias was independently assessed by two researchers according to the PRISMA statement. The disagreements were resolved by discussion.

### 2.4. Statistical Analysis

Meta-analysis was performed using Stata 17 software. Heterogeneity was evaluated using Cochran *Q* test and *I*^2^ test. *I*^2^ > 50% was considered evident heterogeneity. We used the random-effects model for all meta-analysis. Publication bias was assessed by funnel plots and Egger's test. *P* < 0.05 was considered statistically significant.

## 3. Results

**Figure 1:**
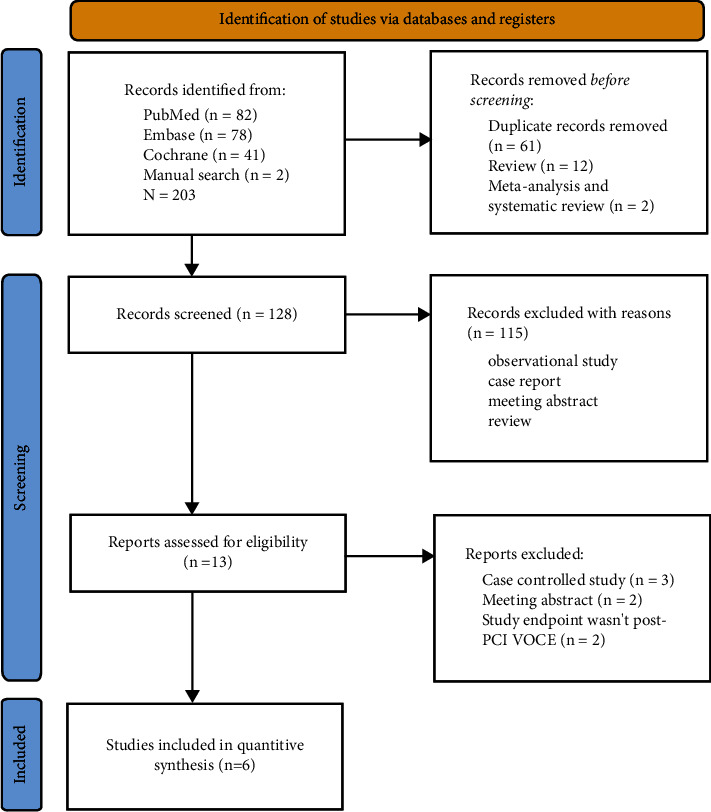
Study selection process.

A total of 203 articles were retrieved: 82 from PubMed, 78 from Embase database, 41 from Cochrane database, and 2 from manual search. After these articles were imported into NoteExpress reference management software (https://www.inoteexpress.com/aegean/), 61 duplicates, 12 reviews, 2 meta-analysis, and systematic reviews were excluded. After reading the abstract of the remaining 128 articles, 115 articles were further excluded. After reading the full text of the remaining 13 articles, 6 articles were finally included in this meta-analysis [1, 12–16]. The flowchart of literature screening is shown in Figure 1.

The characteristics of the included 6 studies were summarized in Table 1. There were 3332 patients involving 4518 target vessels. These studies were published from 2019 to 2022. Four of six studies included in this meta-analysis were from China [13–16], and two of six studies included in this meta-analysis were from other countries [1, 12]. Biscaglia et al.'s study was from Italy and Spain. Kogame et al.'s study was from the Netherlands, Japan, Poland, United Kingdom, Northern Ireland, and Spain. Biscaglia et al.'s [1] study was a registered prospective clinical trial. Three studies, Kogame [12], Zhang [16] and Liu and Ding [15], were retrospective analysis of previously registered prospective clinical trials. Two studies, Tang et al. [13] and Tang [14], were retrospective analysis of previously unregistered prospective data. The follow-up duration was approximately 1-2 years. The PCI type in 4 studies was drug-eluting stent (DES) implantation [1, 12, 13, 16], and the PCI type in the other 2 studies was drug-coated balloon (DCB) expansion [14, 15].

The quality of the included studies was evaluated according to the Newcastle–Ottawa scale (NOS), Table 2. Five studies were of high quality with scores of 8 or 9, and one study was of general quality with a score of 6.

**Figure 2:**
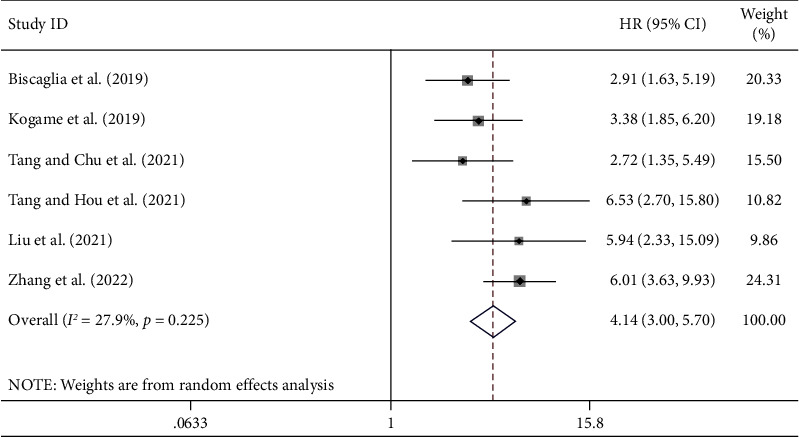
Forest plot (meta-analysis of the hazard ratio of post-PCI VOCE in the lower post-PCI QFR group than in the higher post-PCI QFR group).

**Figure 3:**
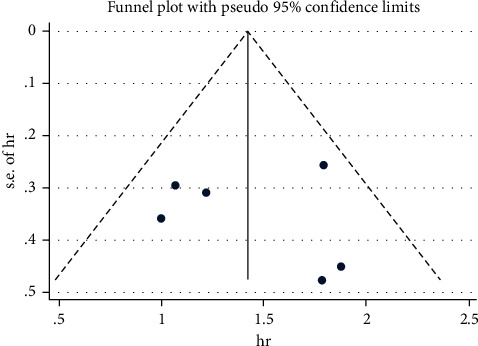
Funnel plots (the hazard ratio of post-PCI VOCE in the lower post-PCI QFR group than in the higher post-PCI QFR group).

The post-PCI QFR cutoff value was not completely same, which ranged from 0.89 to 0.94, Table 3. The target vessel numbers in lower post-PCI QFR group and higher post-PCI QFR group were 1019 and 3499, respectively. The post-PCI VOCE numbers in lower post-PCI QFR group and higher post-PCI QFR group were 168 (16.49%) and 105 (3.00%), respectively. The results of the studies included in this meta-analysis all showed that low post-PCI QFR was an independent risk factor for post-PCI VOCE after adjusting for other factors, HR (95% CI) ranging from 2.718 (1.347–5.486) to 6.53 (2.70–15.8). The factors adjusted for in the 6 studies were as follows: (1) Biscaglia et al.: diabetes, prior MI, lesion length, post-PCI %DS, left anterior descending coronary artery location, and baseline SYNTAX score; (2) Kogame et al.: creatinine clearance, LAD stenosis, and SYNTAX score; (3) Tang and Chu et al.: peak troponin I, diffuse disease, culprit lesion, and diabetes mellitus; (4) Tang and Hou et al.: diabetes mellitus and diameter stenosis (postprocedural in-stent); (5) Liu et al.: diabetes mellitus, difference of drug-coated balloon diameter, and reference vessel diameter (per 0.10-mm increase); (6) Zhang et al.: age, sex, body mass index, hypertension, family history of coronary artery disease, creatine clearance, left ventricular ejection fraction, acute myocardial infarction, vessel SYNTAX score, total occlusion, baseline diameter stenosis, and post-PCI in-stent diameter stenosis. Our meta-analysis showed that the risk of post-PCI VOCE was significantly higher in the lower post-PCI QFR group than in the higher post-PCI QFR group (HR: 4.14, 95% CI: 3.00–5.70, *P* < 0.001, *I*^2^ = 27.9%; Figure 2). Funnel plots were almost symmetric, indicating that there was no evident publication bias, Figure 3. Egger's test also showed no publication bias (*P* = 0.804).

**Figure 4:**
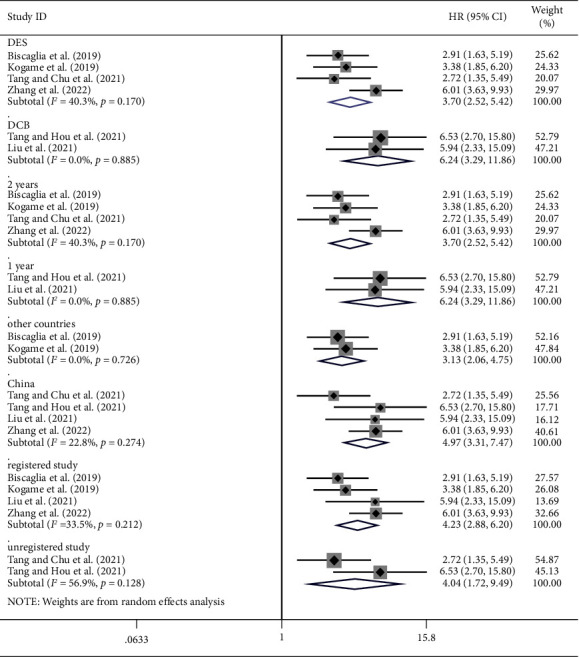
Forest plot (meta-analysis of subgroup).

Subgroup analyses showed that the post-PCI VOCE risk of the drug-eluting stent (DES) subgroup was significantly higher in the lower post-PCI QFR group than in the higher post-PCI QFR (HR: 3.70, 95% CI: 2.52–5.42, *P* < 0.001, *I*^2^ = 40.3%), Figure 4. The post-PCI VOCE risk of the drug-coated balloon (DCB) subgroup was significantly higher in the lower post-PCI QFR group than in the higher post-PCI QFR (HR: 6.24, 95% CI: 3.29–11.86, *P* < 0.001, *I*^2^ = 0.0%). The post-PCI VOCE risk of post-PCI 1 year subgroup was significantly higher in the lower post-PCI QFR group than in the higher post-PCI QFR group (HR: 6.24, 95% CI: 3.29–11.86, *P* < 0.001, *I*^2^ = 0.0%). The post-PCI VOCE risk of post-PCI 2 year subgroup was significantly higher in the lower post-PCI QFR group than in the higher post-PCI QFR group (HR: 3.70, 95% CI: 2.52–5.24, *P* < 0.001, *I*^2^ = 40.3%). The post-PCI VOCE risk in China subgroup was significantly higher in the lower post-PCI QFR group than in the higher post-PCI QFR group (HR: 4.97, 95% CI: 3.31–7.47, *P* < 0.001, *I*^2^ = 22.8%). The post-PCI VOCE risk in other countries subgroup was significantly higher in the lower post-PCI QFR group than in the higher post-PCI QFR group (HR: 3.13, 95% CI: 2.06–4.75, *P* < 0.001, *I*^2^ = 0%). The post-PCI VOCE risk in registered studies subgroup was significantly higher in the lower post-PCI QFR group than in the higher post-PCI QFR group (HR: 4.23, 95% CI: 2.88–6.20, *P* < 0.001, *I*^2^ = 33.5%). The post-PCI VOCE risk in unregistered studies subgroup was significantly higher in the lower post-PCI QFR group than in the higher post-PCI QFR group (HR: 4.04, 95% CI: 1.72–9.49, *P* = 0.001, *I*^2^ = 56.9%).

## 4. Discussion

Since Pijls et al. [20] proposed FFR in 1993, it had become the gold standard for functional evaluation of coronary artery stenosis after long-term basic and clinical research [21]. It involves sending a guidewire with a pressure sensor to the distal end of the coronary artery and measuring the ratio of the pressure at the distal end to the pressure at the proximal end. It requires the use of adenosine, which some patients are intolerant of. Therefore, Tu [11] proposed QFR in 2015, which involves reconstructing the three-dimensional model of the coronary artery and calculating the process of blood flow pressure changes in the whole vessel according to the coronary angiography image. QFR does not require the use of drugs and does not require the use of guidewire. Compared with FFR, QFR has the advantages of simpler operation and better security. Both FFR and QFR belong to functional indicators, which can more accurately reflect the impact of lesions on cardiac function than the pure degree of vascular stenosis. FFR is a direct measurement of pressure and can only be performed during the operation. As long as there is past or present coronary angiography image, QFR can be performed at any time. Therefore, post-PCI QFR can be calculated based on post-PCI coronary angiography images from past prospective databases, and the relationship between post-PCI QFR and long-term prognosis of patients can be analyzed. In this meta-analysis, five studies were retrospective analyses of previous prospective data [12–16], and the other one was a prospective clinical trial [1].

2014 ESC/EACTS Guidelines had clearly stated that it was beneficial to guide PCI based on FFR values measured before PCI [21]. In recent years, studies had shown that QFR and FFR had a very high diagnostic consistency [22, 23]. It was also beneficial to guide PCI based on the QFR values measured before PCI [24]. In addition, it was found that FFR values measured after PCI could predict the long-term outcome of patients [25]. Systematic reviews and meta-analysis had also reached similar conclusions [10, 26]. In recent years, studies had shown that QFR could also predict post-PCI VOCE [1, 12–16]. However, there has been no relevant systematic review and meta-analysis. Therefore, we conducted this meta-analysis, and the results showed that the hazard ratio of post-PCI VOCE was significantly higher in the lower post-PCI QFR group than in the higher post-PCI QFR group.

After successful PCI, many patients still had residual disease, stent underexpansion, and stent edge dissection, which were very important reasons for the occurrence of post-PCI VOCE. Post-PCI QFR measured the stenosis function of target vessels after PCI, which could reflect not only the stented segment but also the nonstented segment. Biscaglia et al.'s [1] study showed that 13% of suboptimal post-PCI QFR was due to PCI segments. For these patients with suboptimal post-PCI QFR, further stent optimization during PCI might could reduce the occurrence of post-PCI VOCE. This meta-analysis showed that mean or median of QFR after angiographically successful PCI were higher than 0.9, and that post-PCI QFR of 5.9% to 7.4% target vessels were still lower than 0.8. Post-PCI QFR could help identify patients at high risk for post-PCI VOCE. In addition, it was noteworthy whether the suboptimal QFR was derived from the PCI segment.

In all 6 studies included in this meta-analysis, post-PCI QFR was used as a binary variable for Cox regression analysis. In addition, post-PCI QFR was also used as a continuous variable for Cox regression analysis in 3 studies. Biscaglia et al.'s [1] study showed that hazard ratio of post-PCI VOCE fell by 0.56 for every 0.1 increase of post-PCI QFR (HR 0.56; 95% CI 0.46–0.68, *P* < 0.001). Tang's [14] study showed that hazard ratio of post-PCI VOCE fell by 0.36 for every 0.1 increase of post-PCI QFR (HR 0.36; 95% CI 0.22–0.59, *P* < 0.001). Liu and Ding's [15] study showed that hazard ratio of post-PCI VOCE fell by 0.34 for every 0.1 increase of post-PCI QFR (HR 0.34; 95% CI 0.23–0.51, *P* < 0.001). These results also showed that the risk of post-PCI VOCE decreases with the increase of post-PCI QFR.

Although the heterogeneity of this meta-analysis was not evident, we still conducted subgroup analyses. The results of subgroup analyses showed that we might need to pay more attention to those who underwent DCB than to those who underwent DES. We might need to pay more attention to the period of post-PCI 1 year than post-PCI 2 year. Biscaglia et al.'s [1] study seems to focus on European participants. Kogame [12] study seems to have diverse ethnical backgrounds. Tang et al.'s [13], Tang's [14], Liu and Ding's [15] and Zhang [16] studies seem to focus on Chinese participants. Post-PCI QFR might have a better predictive value for post-PCI VOCE in Chinese population than other populations. Genetical and cultural implication may be one of the reasons for the difference between Chinese population and other populations. Further investigations are needed to find the pathological cause.

## 5. Limitations

There were some limitations to this meta-analysis. Data recording might be subject to bias, because data of some included studies was from previously unregistered database. In the studies included in this meta-analysis, follow-up time, PCI types, post-PCI QFR cutoff values, and adjusted factors were not completely consistent, which might affect the results of this meta-analysis to some extent. Our meta-analysis would be more convincing if we could obtain the raw data of all studies included in our meta-analysis, reanalyze them, and obtain an optimal cutoff value for post-PCI QFR. To some extent correlation among vessels in a patient may affect results. However, the purpose of our study is to study the predictive value of post-PCI QFR for post-PCI VOCE. Both subjects (post-PCI QFR) and outcomes (post-PCI VOCE) were analyzed based on the number of vessels. This method should be feasible. This is also the case with other meta-analysis, such as the meta-analysis published by Hwang [26] in JAMA in 2022. Hence, the results of this study should be considered exploratory.

## 6. Conclusion

In summary, this meta-analysis showed that post-PCI QFR had a good predictive value for post-PCI VOCE. Large sample prospective studies with carefully controlling for confounders are needed to confirm this conclusion.

## Figures and Tables

**Table 1:** Characteristics of included studies.

Studies	Year	Country	Data source	Follow-up(months)	Patients	Target vessel numbers	Age (years)	Male (%)	Hypertension (%)	Diabetes (%)	Smoke (%)	Hyperlipidemia (%)	LVEF (%)	Target vessel proportion			PCI type
														LAD (%)	LCX (%)	RCA (%)	
Biscaglia et al. [1]	2019	Other countries	HAWKEYE study	21	602	751	68	74	74	23	19	56	NA	47	25	28	DES
Kogame [12]	2019	Other countries	SYNTAX II trial	24	393	771	66.6	92.6	75.4	29.7	14.7	77.1	58.3	45.7	31.5	22.8	DES
Tang et al. [13]	2021	China	Unregistered study	24	186	415	63.1	75.3	61.8	34.9	67.7	18.8	56.2	40.7	25.5	33.7	DES
Tang et al. [14]	2021	China	Unregistered study	12	177	185	68	81	NA	46	NA	NA	NA	50	20	30	DCB
Liu and Ding [15]	2021	China	DCB-ISR trial	12	169	169	62.5	76	73	41	23	34	60.66	48	15	37	DCB
Zhang [16]	2022	China	PANDA III trial	24	1805	2227	60.9	70.2	62	23.9	50.8	32	59.2	48.4	21.6	30	DES

LVEF, left ventricular ejection fraction; LAD, left anterior descending coronary artery; LCX, left circumflex coronary artery; RCA, right coronary artery; PCI, percutaneous coronary intervention; DES, drug-eluting stent; DCB, drug-coated balloon; NA, not available. Biscaglia et al.'s study was from Italy and Spain. Kogame et al.'s study was from the Netherlands, Japan, Poland, United Kingdom, Northern Ireland, and Spain. Hawkeye Study (ClinicalTrials.gov, NCT02811796); SYNTAX II Trial (ClinicalTrials.gov, NCT02015832); PANDA III Trial (ClinicalTrials.gov, NCT02017275).

**Table 2:** Quality evaluation of included studies (the Newcastle–Ottawa scale, NOS).

Studies	Representativeness of the exposed cohort	Selection of the nonexposed cohort	Ascertainment of exposure	Outcomes of interest was not present at start of study	Comparability of cohorts on the basis of the design or analysis	Assessment of outcomes	Follow-up duration was sufficient for outcomes to occur	Adequacy of follow-up of cohorts	Total
Biscaglia et al. [1]	1	1	1	1	2	1	1	1	9
Kogame [12]	1	1	1	1	2	1	1	1	9
Tang et al. [13]	1	1	1	1	2	1	1	0	8
Tang [14]	1	1	1	1	2	0	0	0	6
Liu and Ding [15]	1	1	1	1	2	1	0	1	8
Zhang [16]	1	1	1	1	2	1	1	0	8

The full score is nine.

**Table 3:** Post-PCI QFR value and Cox regression multivariate analysis results.

Studies	Post-PCI QFR mean or median	Post-PCI QFR ≤ 0.8	QFR cutoff	Target vessel numbers		Post-PCI VOCE numbers		HR (95% CI)
				Lower post-PCIQFR group	Higher post-PCIQFR group	Lower post-PCIQFR group (%)	Higher post-PCIQFR group (%)	
Biscaglia et al. [1]	0.97 (0.92, 0.99)	NA	0.89	123	628	31 (25.20%)	22 (3.50%)	2.91 (1.63–5.19)
Kogame [12]	0.91 ± 0.07	7.4%	0.91	284	487	34 (11.97%)	18 (3.70%)	3.38 (1.85–6.20)
Tang et al. [13]	0.94 ± 0.09	NA	0.91	101	314	21 (20.79%)	18 (5.73%)	2.718 (1.347–5.486)
Tang [14]	NA	5.9%	0.94	59	126	20 (33.90%)	7 (5.56%)	6.53 (2.70–15.8)
Liu and Ding [15]	NA	7.1%	0.89	36	133	11 (30.56%)	9 (6.77%)	5.94 (2.33–15.09)
Zhang [16]	0.98 (0.95, 1.00)	NA	0.92	416	1811	51 (12.26%)	31 (1.71%)	6.007 (3.634–9.930)

QFR, quantitative flow ratio; VOCE, vessel-oriented composite endpoint; HR (95% CI), hazard ratio (95% confidence interval); NA, not available. The factors adjusted for in the 6 studies were as follows: (1) Biscaglia et al.: diabetes, prior MI, lesion length, post-PCI %DS, left anterior descending coronary artery location, and baseline SYNTAX score; (2) Kogame et al.: creatinine clearance, LAD stenosis, and SYNTAX score; (3) Tang and Chu et al.: peak troponin I, diffuse disease, culprit lesion, and diabetes mellitus; (4) Tang and Hou et al.: diabetes mellitus, diameter stenosis (post-procedural in-stent); (5) Liu et al.: diabetes mellitus, difference of drug-coated balloon diameter and reference vessel diameter (per 0.10-mm increase); (6) Zhang et al.: age, sex, body mass index, hypertension, family history of coronary artery disease, creatine clearance, left ventricular ejection fraction, acute myocardial infarction, vessel SYNTAX score, total occlusion, baseline diameter stenosis, post-PCI in-stent diameter stenosis.

## Data Availability

All the data generated or analysed during this study are included in this published article.

## Abbreviations

CHD: Coronary atherosclerotic heart disease
IVUS: Intravascular ultrasound
OCT: Optical coherence tomography
FFR: Fractional flow reserve
VOCE: Vessel-oriented composite endpoint
PCI: Percutaneous coronary intervention
QFR: Quantitative flow ratio
DES: Drug-eluting stent
DCB: Drug-coated balloon.

## Supplementary Materials

Supplementary Materials Table 4: occurrences of CV death, TVMI, and TVR 312. Figure 5: Forest plot (CV death, TVMI, and TVR).

## References

[1] Biscaglia S., Matteo T., Salvatore B., Enrico C. (2019). Prognostic value of qfr measured immediately after successful stent implantation. *JACC: Cardiovascular Interventions*.

[2] Piroth Z. (2017). Prognostic value of fractional flow reserve measured immediately after drug-eluting stent implantation. *Circulation: Cardiovascular Interventions*.

[3] Waksman R., Carlo D. M., Rebecca T. (2019). Identification of patients and plaques vulnerable to future coronary events with near-infrared spectroscopy intravascular ultrasound imaging: a prospective, cohort study. *The Lancet*.

[4] Shishikura D. (2020). Progression of ultrasound plaque attenuation and low echogenicity associates with major adverse cardiovascular events. *European Heart Journal*.

[5] Xing L., Higuma T., Wang Z. (2017). Clinical significance of lipid-rich plaque detected by optical coherence tomography: a 4-year follow-up study. *Journal of the American College of Cardiology*.

[6] Kim B. G. (2022). Clinical implications of poststent optical coherence tomographic findings: severe malapposition and cardiac events. *Journal of the American College of Cardiology: Cardiovascular Imaging*.

[7] Agarwal S. K., Kasula S., Hacioglu Y. (2016). Utilizing post-intervention fractional flow reserve to optimize acute results and the relationship to long-term outcomes. *JACC: Cardiovascular Interventions*.

[8] Kasula S. (2016). Clinical and prognostic value of poststenting fractional flow reserve in acute coronary syndromes. *Heart*.

[9] Prati F., Romagnoli E., Burzotta F. (2015). Clinical impact of oct findings during pci: the cli-opci ii study. *Journal of the American College of Cardiology: Cardiovascular Imaging*.

[10] Hakeem A., Uretsky B. F. (2019). Role of postintervention fractional flow reserve to improve procedural and clinical outcomes. *Circulation*.

[11] Tu S. (2015). Fractional flow reserve and coronary bifurcation anatomy: a novel quantitative model to assess and report the stenosis severity of bifurcation lesions. *JACC: Cardiovascular Interventions*.

[12] Kogame N. (2019). Clinical implication of quantitative flow ratio after percutaneous coronary intervention for 3-vessel disease. *JACC: Cardiovascular Interventions*.

[13] Tang J., Chu J., Hou H. (2021). Clinical implication of qfr in patients with st-segment elevation myocardial infarction after drug-eluting stent implantation. *The International Journal of Cardiovascular Imaging*.

[14] Tang J. (2021). Clinical implication of quantitative flow ratio to predict clinical events after drug-coated balloon angioplasty in patients with in-stent restenosis. *Clinical Cardiology*.

[15] Liu L., Ding F. (2023). Prognostic value of post-procedural *μ*qfr for drug-coated balloons in the treatment of in-stent restenosis. *Cardiology Journal*.

[16] Zhang R. (2022). Effects of diabetes mellitus on post-intervention coronary physiological assessment derived by quantitative flow ratio in patients with coronary artery disease underwent percutaneous coronary intervention. *Diabetes Research and Clinical Practice*.

[17] Cai X. (2021). Prognostic value of quantitative flow ratio measured immediately after drug-coated balloon angioplasty for in-stent restenosis. *Catheterization and Cardiovascular Interventions*.

[18] Meng P. N., Liu B. (2021). Cut-off values of lesion and vessel quantitative flow ratio in de novo coronary lesion post-drug-coated balloon therapy predicting vessel restenosis at mid-term follow-up. *Chinese Medical Journal*.

[19] Erbay A. (2021). Prognostic impact of pancoronary quantitative flow ratio assessment in patients undergoing percutaneous coronary intervention for acute coronary syndromes. *Circulation: Cardiovascular Interventions*.

[20] Pijls N. H., van Son J. A., Kirkeeide R. L., De Bruyne B., Gould K. L. (1993). Experimental basis of determining maximum coronary, myocardial, and collateral blood flow by pressure measurements for assessing functional stenosis severity before and after percutaneous transluminal coronary angioplasty. *Circulation*.

[21] Windecker S. (2014). 2014 esc/eacts guidelines on myocardial revascularization: the task force on myocardial revascularization of the european society of cardiology (esc) and the european association for cardio-thoracic surgery (eacts)developed with the special contribution of the european association of percutaneous cardiovascular interventions (eapci). *European Heart Journal*.

[22] Xu B. (2017). Diagnostic accuracy of angiography-based quantitative flow ratio measurements for online assessment of coronary stenosis. *Journal of the American College of Cardiology*.

[23] Westra J., Andersen B. K. (2018). Diagnostic performance of in-procedure angiography-derived quantitative flow reserve compared to pressure-derived fractional flow reserve: the favor ii europe-Japan study. *Journal of the American Heart Association*.

[24] Xu B. (2021). Angiographic quantitative flow ratio-guided coronary intervention (favor iii China): a multicentre, randomised, sham-controlled trial. *The Lancet*.

[25] Hoshino M. (2019). Prognostic value of post-intervention fractional flow reserve after intravascular ultrasound-guided second-generation drug-eluting coronary stenting. *Eurointervention*.

[26] Hwang D. (2022). Prognostic implications of fractional flow reserve after coronary stenting: a systematic review and meta-analysis. *JAMA Network Open*.

