# Supplementary material for: Predictive Value of Post-Percutaneous Coronary Intervention Quantitative Flow Ratio for Vessel-Oriented Composite Endpoint
Source: J Interv Cardiol. 2023 Sep 9;2023:2438347. doi: 10.1155/2023/2438347 (PMC10505082; doi:10.1155/2023/2438347)
Supplement: Supplementary Materials — Table 4: occurrences of CV death, TVMI, and TVR 312. Figure 5: Forest plot (CV death, TVMI, and TVR). [file 2438347.f1.zip › Table 4 Occurrences of CV death, TVMI and TVR (1).docx]

| **Table 4 Occurrences of CV death, TVMI and TVR** | | | | | | |
| --- | --- | --- | --- | --- | --- | --- |
| study | CV death | | TVMI | | TVR | |
|  | Lower post-PCI QFR group | Higher post-PCI QFR group | Lower post-PCI QFR group | Higher post-PCI QFR group | Lower post-PCI QFR group | Higher post-PCI QFR group |
| Biscaglia et al. (1) | 9 | 7 | 10 | 11 | 24 | 16 |
| Kogame et al. (12) | 4 | 4 | 4 | 4 | 30 | 14 |
| Tang and Chu et al. (13) | NA | NA | NA | NA | NA | NA |
| Tang and Hou et al. (14) | 1 | 0 | 2 | 1 | 19 | 6 |
| Liu et al. (15) | 1 | 0 | 2 | 0 | 10 | 9 |
| Zhang et al. (16) | 15 | 11 | 9 | 10 | 33 | 15 |
| VOCE, vessel-oriented composite endpoint; QFR, quantitative flow ratio; CV, cardiovascular; TVMI, target vessel myocardial infarction; TVR, target vessel revascularization; NA, not available. | | | | | | |
