# Supplementary figures and images for: Predictive Value of Post-Percutaneous Coronary Intervention Quantitative Flow Ratio for Vessel-Oriented Composite Endpoint
Source: J Interv Cardiol. 2023 Sep 9;2023:2438347. doi: 10.1155/2023/2438347 (PMC10505082; doi:10.1155/2023/2438347)

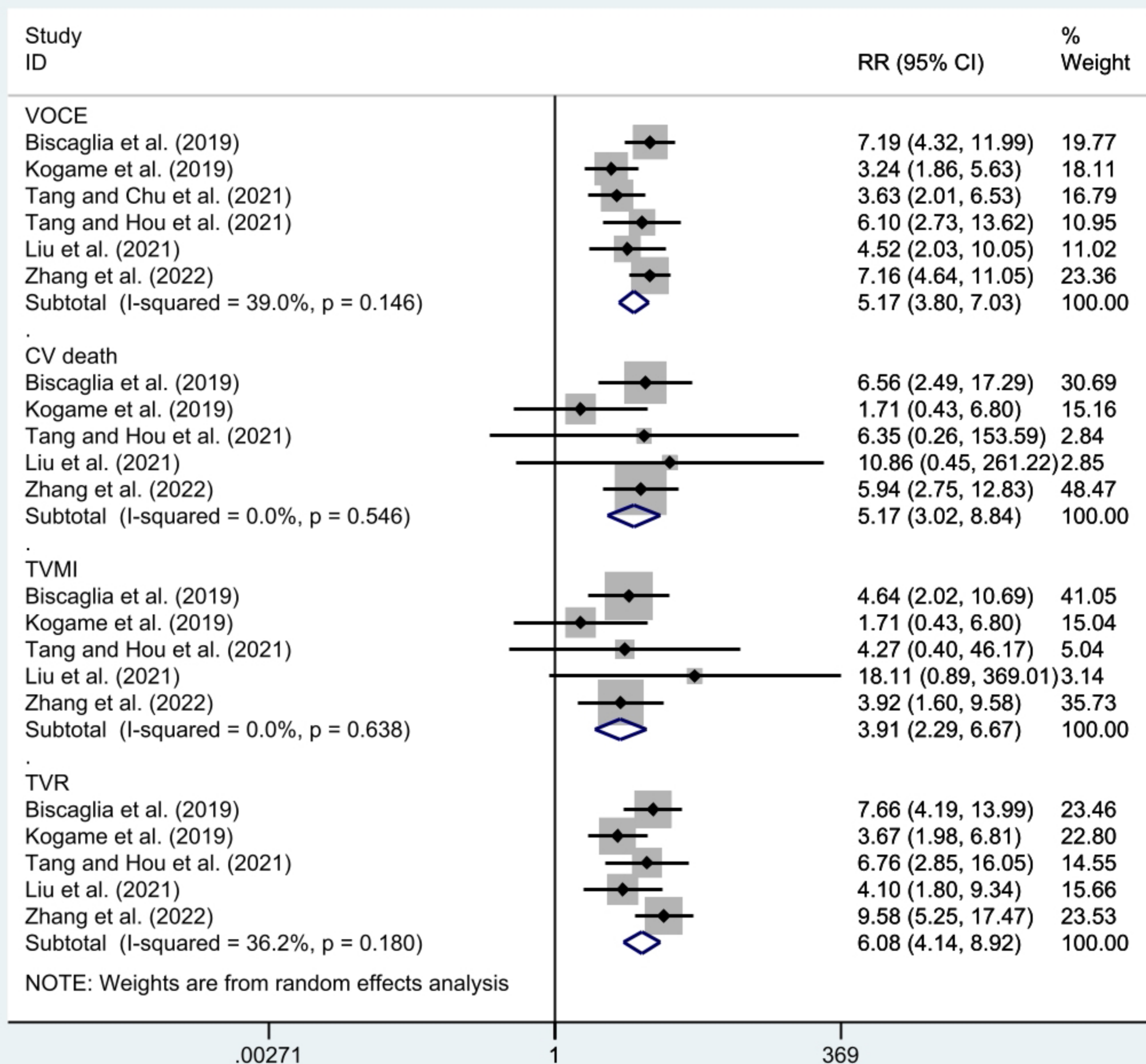

Supplement: Supplementary Materials — Table 4: occurrences of CV death, TVMI, and TVR 312. Figure 5: Forest plot (CV death, TVMI, and TVR). [file 2438347.f1.zip › Figure 5 Forest plot(CV death, TVMI and TVR).pdf]
